# Chromoblastomycosis in an Immunocompetent Child: A Case Report

**DOI:** 10.7759/cureus.96406

**Published:** 2025-11-09

**Authors:** Mohib Ullah, Muhammad Khurram Shahzad, Anum Mushtaq Khan, Erum Ramzan

**Affiliations:** 1 Department of Dermatology, Sheikh Zayed Medical College & Hospital, Rahim Yar Khan, PAK

**Keywords:** antifungal agents, chromoblastomycosis, drug resistance, drug therapy, fungal, immunocompetent, multiple, therapeutic use

## Abstract

Chromoblastomycosis is a chronic cutaneous infection caused by dematiaceous pigmented fungi. Lesions may begin as an erythematous papule, which progresses to a hyperkeratotic plaque. Management of chromoblastomycosis is usually challenging. We report a case of a 12-year-old immunocompetent male child with chromoblastomycosis, characterized by warty, hyperkeratotic plaques on the face. The diagnosis was made by histopathology and clinical examination. Histopathology confirmed the presence of a brownish, dematiaceous, fungal organism with internal septations. The patient was treated with oral itraconazole and terbinafine for six months.

## Introduction

Chromoblastomycosis or chromomycosis is a pigmented, chronic infection of the skin and subcutaneous tissue caused by fungi [1]. It is a slow-growing fungal infection that begins as a small firm, red or grey bump that progresses to small nodules, eventually evolving into a warty plaque. Sometimes, the condition can become painful, leading to complications such as cellulitis and secondary bacterial infections. The cases of chromoblastomycosis are mainly reported in tropical areas, where the efficacy of medicine and treatment options are limited. The World Health Organization (WHO) has included chromoblastomycosis as one of the neglected tropical diseases (NTDs) [2]. ​​​​​​Treatment options for chromoblastomycosis are multiple, such as antifungal treatment, cryotherapy, and surgery. Antifungal itraconazole and terbinafine have shown good results [3].

## Case presentation

**Figure 1:**
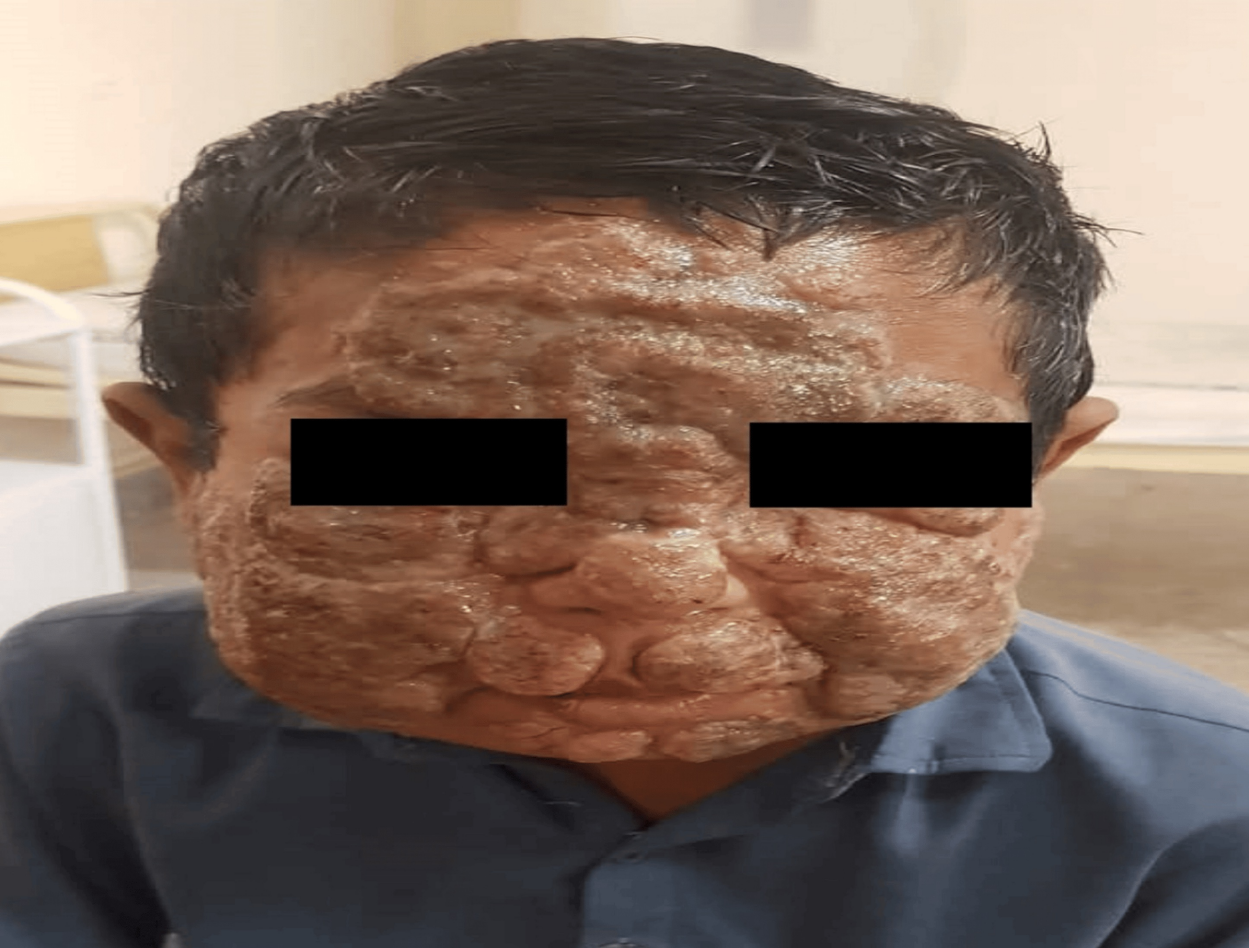
Lesions on the face. Image showing the child with verrucous and hyperkeratotic plaques.

We present the case of a 12-year-old child who attended a dermatological consultation due to lesions on his face for the last one year. There was no personal or family history of dermatological illness. The lesion was characterized by painless, verrucous, and hyperkeratotic plaques. The lesion involved the whole face, including the forehead, nose, both cheeks, and jaws. Initially, the lesion started as skin color papules, which gradually progressed to a verrucous plaque. There was no history of pain, fever, or itching (Figure 1).

He received treatment from various hospitals, but there was no satisfactory response to various therapies. Physical examination did not show any systemic abnormality. Examination of the face revealed hyperkeratotic verrucous plaques involving the whole face. Laboratory investigations, including hepatitis B and C and HIV screening tests, were all negative. X-ray of the chest in posteroanterior (PA) view and ultrasound of his abdomen did not show any abnormality.

**Figure 2:**
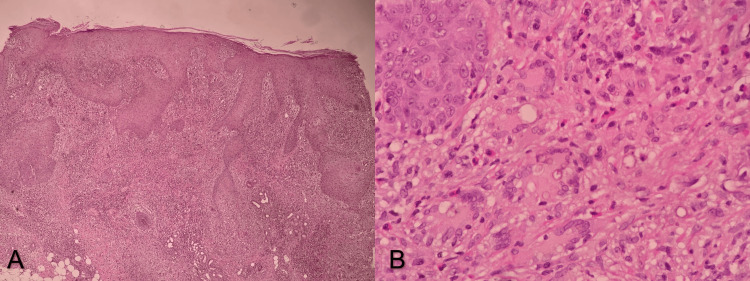
Histopathology of the lesion with epidermis and dermis. (A) Low-power image showing epidermal hyperplasia and intense dermal infiltrate. (B) High-power image showing mixed dermal infiltrate and numerous brownish, dematiaceous, and fungal organisms with internal septations.

Punch biopsy was performed, and histopathology of the skin showed hyperkeratosis, parakeratosis, acanthosis, spongiosis, and exocytosis. At some places, neutrophils were identified in the stratum corneum layer of the skin. Dermis showed collections of epithelioid cells and multinucleated giant cells, along with interstitial perivascular and periadnexal mixed inflammation, comprising lymphocytes, histiocytes, plasma cells, neutrophils, and eosinophils. Necrosis was also present on histopathology. Numerous brownish, dematiaceous, fungal organisms with internal septations were seen. Periodic acid-Schiff with diastase (PASD) stain was positive for a fungal organism (Figure 2).

**Figure 3:**
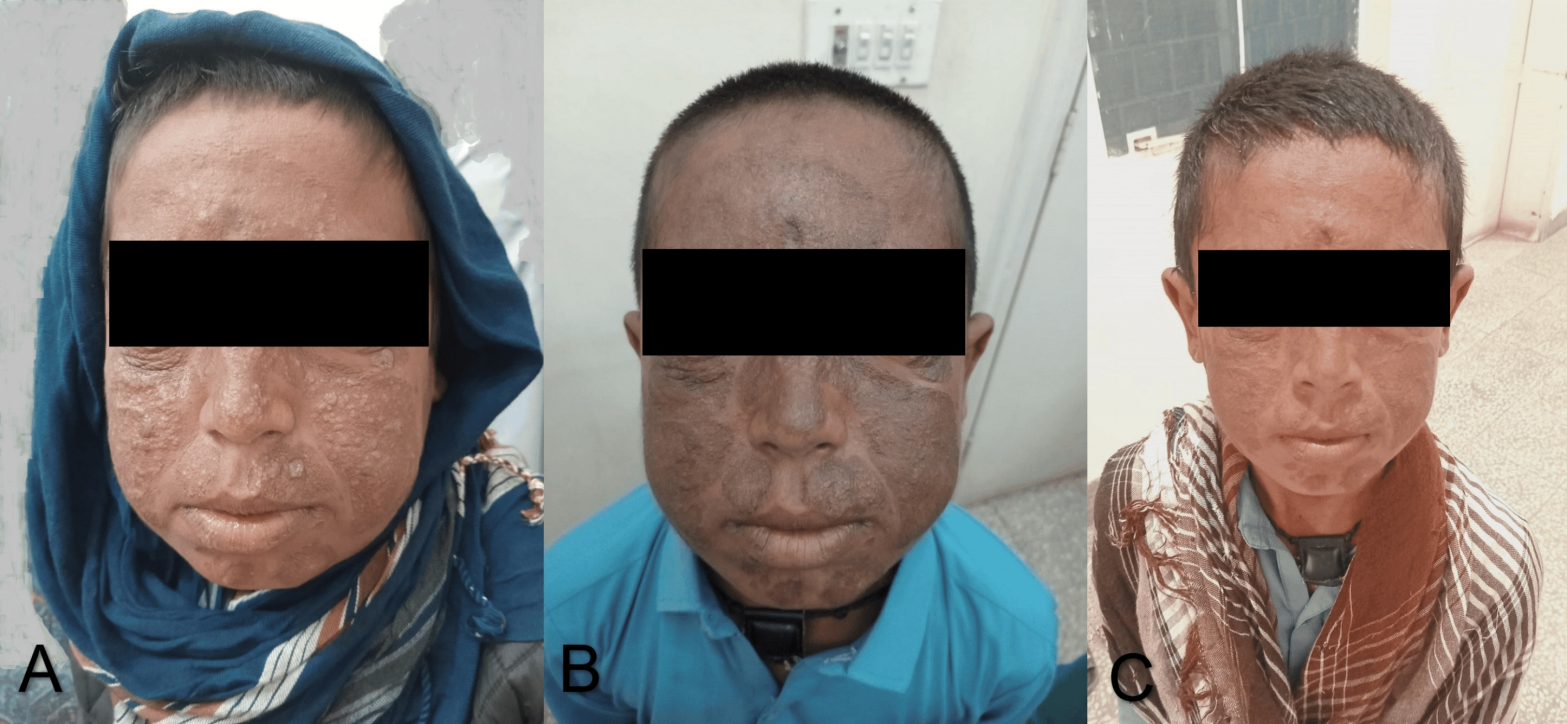
Post-treatment appearance showing significant clinical improvement. (A) After two months of therapy, the plaque decreased in size, and there were minimal hyperkeratotic lesions. (B) Image after four months of therapy showing the lesion with no hyperkeratotic plaque present. (C) Image after six months of treatment showing only post-inflammatory hyperpigmentation, with no sign of hyperkeratotic plaque.

The findings and clinical examination confirmed the diagnosis of chromoblastomycosis. Treatment with itraconazole (200 mg/day) and terbinafine (250 mg/day) was started. The patient was called for follow-up every two months. A complete clearance of the disease was noted at the end of six months of therapy (Figure 3).

## Discussion

Chromoblastomycosis is a chronic, slowly progressive, subcutaneous mycosis, mostly reported from temperate climates. It is more prevalent in people who belong to temperate and tropical regions, having occupations related to agriculture, such as farmers, gardeners, and woodworkers. Patients often have a history of some skin wound due to penetration of a splinter, wood, or thorn [4]. *Fonsecaea pedrosoi* and *Cladophialophora carrionii* are the most common etiologic agents. The lesions resemble various dermatological diseases. Therefore, the diagnosis relies on detecting muriform cells in tissue and confirming the causal agent through culture. Diagnosis can be confirmed by potassium hydroxide (KOH) preparation or biopsy, which reveals muriform cells (sclerotic bodies). Histopathology shows hyperkeratosis, pseudoepitheliomatous hyperplasia, and granulomas with Langerhans giant cells [5].

Chromoblastomycosis mainly affects the peripheries, such as the feet, legs, and hands, which often follow inoculation of organisms after a micro- or macro-traumatic wound. Cases involving the face, buttock, ear, and trunk have been reported in the literature [6]. The initial lesion involves an erythematous macule that later on progresses to papular and papulosquamous forms, giving a polymorphic presentation that often mimics other dermatological conditions [7]. Pruritus is the main symptom [8]. The infection mostly remains confined to the subcutaneous tissues and slowly progresses. It can lead to fibrosis, recurrent bacterial infections, chronic lymphedema, elephantiasis, and squamous cell carcinoma in advanced cases [9].

The clinical findings of our case, the presentation, and treatment protocol, including response to therapy, add to the existing literature. The treatment for chromoblastomycosis is divided into three main groups, which include medical therapy, physical modalities, and chemotherapy. For medical therapy, a combination of various antifungal medications, such as itraconazole and terbinafine, is used for six to 12 months [5]. The use of a single drug therapy has been reported with the development of resistance and less effective response [10]. Surgery is an option in case of recurrence. However, in some cases, the spread is so extensive that a wide surgical excision is not possible [11].

## Conclusions

Chromoblastomycosis is a persistent, progressive fungal infection that is challenging to eradicate. For prompt management and enhancing treatment results, early diagnosis with KOH skin preparation or biopsy is essential. The treatment based on culture, using multiple drugs, is necessary along with regular follow-ups and serial biopsies to ensure complete eradication and minimal relapse risk. Given the disease’s neglected status and its potential to mimic other dermatologic conditions, increased clinical awareness and further research into optimized treatment strategies are necessary, particularly for advanced and resistant cases.
